# Detection of asymptomatic coronary artery disease among patients with type 2 diabetes mellitus using treadmill test

**DOI:** 10.6026/973206300214128

**Published:** 2025-11-15

**Authors:** Gagan Gunjan, Ritesh L Chauhan, Pushpendra Singh Sengar, Madhur Sharma

**Affiliations:** 1 Department of Medicine, Rajendra Institute of Medical Sciences, Ranchi, Jharkhand, India; 2 Department of General Medicine, RD Gardi Medical College, Ujjain, Madhya Pradesh, India; 3 Department of Medicine, Bundelkhand Medical College & Hospital, Sagar, Madhya Pradesh, India; 4 Department of Gastroenterology, MMIMSR, Ambala, Haryana, India

**Keywords:** Type 2 diabetes mellitus, asymptomatic coronary artery disease, treadmill test, silent myocardial ischemia, HbA1c, dyslipidemia

## Abstract

Type 2 diabetes mellitus (T2DM) is strongly associated with silent myocardial ischemia (SMI), an asymptomatic form of coronary artery disease (CAD) and making early detection vital to prevent cardiac events. Therefore, it is of interest to evaluate 75 asymptomatic T2DM patients (mean age 53.27 ± 9.22 years) using the treadmill test (TMT) with the Bruce protocol and compared clinical and biochemical parameters between TMT-positive and negative groups. TMT was positive in 20 patients (26.66%), who were significantly older and had longer diabetes duration, higher HbA1c, fasting and postprandial glucose levels and adverse lipid profiles compared to TMT-negative patients. Dyslipidemia, elevated triglycerides and LDL and reduced HDL were particularly prominent among TMT-positive individuals. Thus, we show that longer disease duration, poor glycemic control and dyslipidemia are strong predictors of asymptomatic CAD and TMT is a useful non-invasive tool for its early detection in T2DM patients.

## Background:

With an estimated 463 million people afflicted in 2019 and predictions that the number will increase to 642 million by 2040, diabetes mellitus has reached epidemic proportions worldwide [1]. About 73 million people in India, dubbed the "diabetic capital of the world," had diabetes in 2015; estimates indicate that by 2045, this will rise to 134 million [2]. 90-95% of all instances of diabetes are type 2 diabetes mellitus (T2DM), which is distinguished by elevated hepatic glucose production, insulin resistance and relative insulin insufficiency [3]. The primary cause of death for those with type 2 diabetes is cardiovascular disease, with coronary artery disease (CAD) being especially common [4]. According to the Framingham Heart Study, the cardiovascular mortality rate for diabetic women is four times higher than that of their non-diabetic counterparts and it is twice as high for diabetic males [5]. Additionally, silent myocardial ischaemia (SMI), a common asymptomatic presentation of CAD in diabetic individuals, might cause delayed diagnosis and treatment, raising the risk of acute cardiac events [6]. Diabetes-related accelerated atherosclerosis has a complex pathogenesis that includes inflammation, endothelial dysfunction, insulin resistance, hyperglycemia and dyslipidaemia [7]. Vascular injury is facilitated by the production of advanced glycation end products (AGEs), which are encouraged by hyperglycemia [8]. Cardiovascular risk is further increased by diabetic dyslipidaemia, which is typified by small, dense LDL particles, low HDL cholesterol and excessive triglycerides [9].

In people with diabetes, the occurrence of silent myocardial ischemia (SMI) is much more common compared to those without diabetes, with some studies showing rates between 21% and 37% [10, 11]. This happens partly because of a condition called cardiac autonomic neuropathy, which makes it harder for the body to feel chest pain during a heart attack [12]. Finding SMI early in people with diabetes who don't have symptoms is very important, as it helps start treatments early and avoids serious heart problems. There are several methods to check for CAD without doing any surgery. These include ECG while resting, stress echocardiography, imaging of the heart muscle blood flow and treadmill tests during exercise [13]. Among these, the treadmill test is commonly used, affordable and easy to get, especially in places where there are not many resources [14]. Even though many studies have looked at how often SMI happens in T2DM patients using treadmill tests, there is not much information about this in India. In India, people get diabetes at a younger age and have different health problems compared to people in Western countries [15]. Therefore, it is of interest to evaluate the detection of asymptomatic coronary artery disease in patients with type 2 diabetes mellitus using treadmill test.

## Materials and Methods:

## Study design and setting:

This observational study was conducted at the Department of Medicine, Shri Aurobindo Medical College & Post Graduate Institute, Indore, over a period of 18 months (October 2018 to May 2020).

## Study population:

A total of 75 patients (57 males and 18 females) with T2DM diagnosed according to the American Diabetes Association (ADA) criteria were included in the study. Both inpatients and outpatients attending the hospital during the study period were considered for enrollment.

## Inclusion criteria:

[1] Patients with T2DM diagnosed by ADA criteria (HbA1c >6.5%, FBS >125 mg/dl, or PPBS >200 mg/dl)

[2] Age between 18 and 70 years

[3] Patients providing written informed consent to participate in the study

## Exclusion criteria:

[1] Individuals with pre-existing congenital heart disease, known CAD, or valvular heart disease

[2] Patients with uncontrolled hypertension or unstable angina

[3] Patients with physical disabilities (e.g., severe osteoarthritis) are prevented from performing the treadmill test

[4] Patients with anemia (haemoglobin <8 g %)

[5] Patients who did not provide written consent for the study

## Sample size and sampling technique:

A selective sampling technique was used to enroll 75 T2DM patients who underwent treadmill testing during the study period.

## Methodology:

## Patient assessment:

## Patient assessment:

All participants underwent a comprehensive evaluation, including:

[1] Detailed medical history focusing on duration of diabetes, cardiovascular risk factors and symptoms

[2] Physical examination, including measurement of height, weight, blood pressure and cardiovascular examination

[3] Laboratory investigations:

1) Complete blood count

2) Fasting and postprandial blood glucose (FBS and PPBS)

3) Glycosylated hemoglobin (HbA1c) measured using High Performance Liquid Chromatography

4) Lipid profile (total cholesterol, HDL, LDL, triglycerides)

5) Renal function tests (creatinine, SGPT, SGOT)

[4] Baseline 12-lead ECG

[5] 2D echocardiography using the Phillips iE33 machine

## Treadmill test protocol:

All patients underwent a treadmill test using the GE MAC 5500 machine following the Bruce protocol.

The procedure was performed according to standardized guidelines:

[1] Patients were instructed to avoid eating, smoking, or consuming caffeine for at least 3 hours before the test.

[2] After obtaining informed consent, a baseline 12-lead ECG and blood pressure measurement were recorded.

[3] Patients were shown the treadmill walking technique before beginning the test.

[4] The Bruce protocol was initiated, with progressive increases in speed and grade every 3 minutes.

[5] Continuous ECG monitoring was performed throughout the test, with 12-lead ECG recordings obtained at the end of each stage.

[6] Blood pressure was measured at the end of each stage and during the recovery period.

[7] The test was terminated upon achievement of the target heart rate (85% of maximum predicted heart rate, calculated as 220 - age in years) or if any of the following occurred:

1) Development of typical anginal pain

2) ST-segment depression ≥1 mm horizontal or downsloping

3) ST-segment elevation ≥1 mm

4) Significant arrhythmias

5) Hypotension (systolic blood pressure drop >20 mmHg)

6) Severe dyspnea, fatigue, or dizziness

7) Patient's request to stop

## Interpretation of TMT results:

A positive TMT result was defined as the development of ≥1 mm horizontal or down sloping ST-segment depression 80 ms after the J-point in three consecutive beats, compared with the resting ECG. All TMT results were interpreted by experienced cardiologists who were blinded to the patients' clinical and laboratory data.

## Statistical analysis:

Data were analyzed using SPSS statistical software (version 22.0).

## Results:

A total of 75 T2DM patients (57 males, 18 females) with a mean age of 53.27 ± 9.22 years (range: 28-70 years) were included in the study. The demographic and clinical characteristics of the study population are presented in Table 1 (see PDF). TMT was positive in 20 patients (26.66%) and negative in 55 patients (73.34%). Among the TMT-positive patients, 15 (75%) were male and 5 (25%) were female. The mean age of TMT-positive patients was significantly higher than that of TMT-negative patients (60.05 ± 5.02 vs. 50.80 ± 9.27 years, p < 0.0001). Regarding the duration of diabetes, none of the patients with a diabetes duration <5 years had a positive TMT result. Among patients with diabetes duration of 5-10 years, 13 (40.6%) had a positive TMT result, while 7 (70%) of those with diabetes duration >10 years had a positive TMT result (p < 0.0001). The metabolic parameters of patients with positive and negative TMT results are compared in Table 2 (see PDF). The distribution of TMT results across different age groups is presented in Table 3 (see PDF). None of the patients in the 28-37 years and 38-47 years age groups had a positive TMT result. In the 48-57 years age group, 22.6% (7 out of 31) had a positive TMT result, while in the >58 years age group, 48.1% (13 out of 27) had a positive TMT result (p = 0.004).

## Discussion:

The present study aimed to detect asymptomatic CAD in patients with T2DM using TMT and to evaluate its association with disease duration, glycemic control and other cardiovascular risk factors. Our findings revealed that 26.66% of asymptomatic T2DM patients had a positive TMT result, indicating the presence of silent myocardial ischemia. The prevalence of SMI in our study (26.66%) is consistent with previous research. Ahuja and others [16] found that 21.1% 161 people with type 2 diabetes who didn't have symptoms had CAD. Zakaviand and others [17] found that 24% of their group of asymptomatic type 2 diabetes patients had CAD. Similarly, Sharma *et al.* (2021) reported a 26.66% prevalence of asymptomatic coronary artery disease among Indian patients with type 2 diabetes mellitus using treadmill testing, reinforcing the comparable magnitude of silent ischemia observed in our study [18]. These results show that a lot of people with diabetes may have CAD without knowing it, which shows how important it is to do regular check-ups. Our study showed a strong link between age and the presence of SMI. Patients who presented with positive TMT were much older, averaging about 60.05 years with a standard deviation of 5.02, compared to those with negative results, who averaged around 50.80 years with a standard deviation of 9.27 (p < 0.0001) [19]. The present result matches with earlier research showing, getting older is a big risk factor for CAD in people with diabetes [20, 21]. The higher rate of SMI in older diabetic patients could be because they have been exposed to high blood sugar for longer, have more advanced glycation end products built up and experience age-related problems with blood vessel function [22]. The duration of diabetes emerged as another significant predictor of SMI in our study. None of the patients with diabetes duration <5 years had a positive TMT result, whereas 40.6% of those with diabetes duration of 5-10 years and 70% of those with duration >10 years had positive TMT results (p < 0.0001). This finding is consistent with previous research by Lau *et al.* [23], who reported a significantly longer duration of diabetes in TMT-positive patients compared to TMT-negative patients (4.91 ± 2.51 years vs. 3.59 ± 2.13 years). The association between diabetes duration and CAD risk may be explained by the cumulative exposure to hyperglycemia and its detrimental effects on the vascular endothelium over time [24]. Poor glycemic control, as reflected by elevated HbA1c levels, was significantly associated with SMI in our study. Patients with positive TMT results had significantly higher HbA1c levels (9.82 ± 1.35%) compared to those with negative results (8.07 ± 1.34%, p < 0.0001). This finding is consistent with previous research that has demonstrated a strong association between poor glycemic control and increased risk of CAD in diabetic patients [25]. Chronic hyperglycemia promotes the formation of advanced glycation end products, oxidative stress, inflammation and endothelial dysfunction, all of which contribute to the development and progression of atherosclerosis [26]. Dyslipidemia was another significant predictor of SMI in our study. Patients with positive TMT results had significantly higher levels of total cholesterol, LDL and triglycerides and lower levels of HDL compared to those with negative results. This finding is consistent with previous research that has identified diabetic dyslipidemia as a major risk factor for CAD [27, 28]. The characteristic dyslipidemia in T2DM, characterized by elevated triglycerides, low HDL cholesterol and small, dense LDL particles, is highly atherogenic and contributes to the accelerated development of CAD in diabetic patients [29]. The findings of our study have important clinical implications. The high prevalence of asymptomatic CAD in T2DM patients highlights the importance of regular cardiovascular screening in this high-risk population. TMT, being a non-invasive, widely available and cost-effective test, can serve as a valuable screening tool for detecting SMI in asymptomatic T2DM patients. Early detection of SMI allows for timely intervention with risk factor modification, anti-ischemic medications and, when indicated, revascularization procedures, potentially preventing adverse cardiac events and improving outcomes. Our study has several limitations. First, the sample size was relatively small, which may limit the generalizability of our findings. Second, we used TMT as the sole diagnostic tool for detecting SMI, which has lower sensitivity and specificity compared to more advanced imaging techniques such as stress echocardiography or myocardial perfusion imaging. Third, we did not perform coronary angiography to confirm the presence and severity of CAD in patients with positive TMT results. Fourth, the cross-sectional design of our study does not allow for establishing causality or determining the long-term prognostic significance of SMI in T2DM patients. Future research should include larger prospective studies with longer follow-up periods to determine the prognostic significance of SMI detected by TMT in T2DM patients. Additionally, studies comparing the diagnostic accuracy of TMT with other non-invasive imaging modalities in detecting SMI in diabetic patients would be valuable.

## Conclusion:

A high prevalence (26.66%) of asymptomatic CAD in patients with T2DM, as detected by TMT is shown. Longer duration of diabetes, poor glycemic control (elevated HbA1c) and dyslipidemia were significant predictors of silent myocardial ischemia. TMT serves as a valuable, non-invasive and cost-effective screening tool for detecting asymptomatic CAD in T2DM patients.
